# Sudden cardiac death athletes: a systematic review

**DOI:** 10.1186/1758-2555-2-19

**Published:** 2010-08-03

**Authors:** Marcelo Ferreira, Paulo Roberto Santos-Silva, Luiz Carlos de Abreu, Vitor E Valenti, Vanessa Crispim, Caio Imaizumi, Celso Ferreira Filho, Neif Murad, Adriano Meneghini, Andrés R Pérez Riera, Tatiana Dias de Carvalho, Luiz Carlos Marques Vanderlei, Erica E Valenti, José R Cisternas, Oseas F Moura Filho, Celso Ferreira

**Affiliations:** 1 Departamento de Clínica Médica, Disciplina de Cardiologia, Núcleo de Saúde no Esporte, Faculdade de Medicina do ABC, Santo André, SP, Brasil; 2 Departamento de Morfologia e Fisiologia, Faculdade de Medicina do ABC, Santo André, SP, Brasil; 3 Departamento de Medicina, Disciplina de Cardiologia, Universidade Federal de São Paulo (UNIFESP), São Paulo, SP, Brasil; 4 Departamento de Fisioterapia, Universidade Estadual Paulista, Campus de Presidente Prudente, Presidente Prudente, SP, Brasil; 5 Departamento de Educação Física e Motricidade Humana, Universidade Federal de São Carlos, São Carlos, SP, Brasil

## Abstract

Previous events evidence that sudden cardiac death (SCD) in athletes is still a reality and it keeps challenging cardiologists. Considering the importance of SCD in athletes and the requisite for an update of this matter, we endeavored to describe SCD in athletes. The Medline (via PubMed) and SciELO databases were searched using the subject keywords "sudden death, athletes and mortality". The incidence of SCD is expected at one case for each 200,000 young athletes per year. Overall it is resulted of complex dealings of factors such as arrhythmogenic substrate, regulator and triggers factors. In great part of deaths caused by heart disease in athletes younger than 35 years old investigations evidence cardiac congenital abnormalities. Athletes above 35 years old possibly die due to impairments of coronary heart disease, frequently caused by atherosclerosis. Myocardial ischemia and myocardial infarction are responsible for the most cases of SCD above this age (80%). Pre-participatory athletes' evaluation helps to recognize situations that may put the athlete's life in risk including cardiovascular diseases. In summary, cardiologic examinations of athletes' pre-competition routine is an important way to minimize the risk of SCD.

## Background

Athlete is defined as who participates in individual or collective sports (any age) as well as training programs of systematic and regular competitions [1-7].

Recent events in all parts of the world show that sudden cardiac death (SCD) of athletes is still a reality and it continues to challenge experts in cardiology which works with athletes [8-13]. SCD is defined by the World Health Organization (WHO) as that condition which occurs within the first 24 hours after the symptoms onset. However, several experts decrease this time and describe it as an unexpected death, marked by abrupt loss of consciousness in an individual within the first hour after symptoms onset with or without heart disease [14].

SCD in athletes is rare (1:50,000 - 1:100,000 annually), however, it is still 2-4 times more often when compared to non athletes. Several mechanisms are proposed: myocardial ischemia, repolarization due to potassium channel downregulation and it may also be explained by the presence of multiple factors such as cardiac hypertrophy (athlete's heart) and/or hypertrophic cardiomyopathy, increased sympathetic tone, genetic defects, drugs, doping agents or food ingredients. These factors together may increase the repolarization homogeneity which sometimes leads to risk of arrhythmias and SCD [15].

In not selected population the incidence of SCD is 0.001-0.002% per year. Nevertheless, in absolute value it generates a significantly higher number. In selected populations such as athletes the absolute value of deaths decreased but the relative risk increases [15].

In Brazil and in the rest of the Latin American continent, the only statistic of SCD tracked over the years is from the Institute of Cardiology Dante Pazzanese. More than 25 years they have monitored SCD in athletes. The statistics of this institution shows that in children and adolescents mild abnormalities risks ranged from 17.7 to 21.0% while in active amateur athletes and in professionals younger than 35 years old it was 8.2%, this only for cardiac factors. The results of the Italian Olympic Committee regarding removal of athletes for heart disease were only 3% [16].

We believe that cardiologic evaluation of athletes' pre-competition routine is the only way to minimize the risk of SCD in this selected population. In view of the importance of SCD in athletes and the necessity for an update of this issue, we aimed to describe SCD in athletes through a systematic review.

## Method

The Medline (via PubMed) and SciELO databases were searched using the subject keywords "sudden death, athletes and mortality". We also used the "related articles" function on PubMed and SciELO with the references of the studies. Publications were included in our review if their titles or abstracts were available in English or Portuguese and described SCD in athletes. The review was completed on June 2010. Publications were excluded if the treatment was limited to a particular technique.

Each publication was reviewed in order to identify the author(s), study period and data source.

## Results

In the review which we made in the mentioned databases we found a total of 694 manuscripts. We excluded investigations where the management was limited to a particular method.

We summarized the main studies in Table 1, which briefly describes important studies regarding SCD in athletes.

**Table 1:** Summary of the main clinical studies regarding SCD in athletes.

*Author and year*	*Main findings*
Germann et al, 2005 [2].	Most etiologies of SCD in athletes result in the same final common denominator (cardiac arrest) on presentation to an emergency physician. There are certain historic, physical examination, and electrocardiographic features of many of these disease processes that emergency physicians should have a working knowledge of to try to identify them before they result in SCD.
Corrado et al, 2006 [3].	The incidence of SCD in young competitive athletes has substantially declined in the Veneto region of Italy since the introduction of a nationwide systematic screening. Mortality reduction was predominantly due to a lower incidence of sudden death from cardiomyopathies that paralleled the increasing identification of athletes with cardiomyopathies at preparticipation screening.
Corrado et al, 2003 [15].	Sports activity in adolescents and young adults was associated with an increased risk of SCD, both in males and females. Sports was not a cause of the enhanced mortality, but it triggered SCD in those athletes who were affected by cardiovascular conditions predisposing to life-threatening ventricular arrhythmias during physical exercise
Maron et al, 1986 [46].	In most young competitive athletes (less than 35 years of age) sudden death is due to congenital cardiovascular disease. Hypertrophic cardiomyopathy appears to be the most common cause of such deaths (about half of the sudden deaths in young athletes). Other cardiovascular abnormalities that appear to be less frequent but important causes of sudden death in young athletes include congenital coronary artery anomalies, ruptured aorta (due to cystic medial necrosis), idiopathic left ventricular hypertrophy and coronary artery atherosclerosis. Diseases that appear to be very uncommon causes of sudden death include myocarditis, mitral valve prolapse, aortic valve stenosis and sarcoidosis.

SCD: Sudden Cardiac Deaths.

## Discussion

### Epidemiology

The prevalence of SCD is estimated in one case for each 200,000 young athletes [17-19]. On the other hand, the occurrence of SCD associated to training and competitions for athletes is increased by 2.8 times compared to the average relative risk of non-competitive practitioners [20], hence giving rise to the following question: Does sports activity causes sudden death in young people?

The most common cause of sudden death is cardiovascular origin according to the literature, with incidence and nature of the subject areas. For example, the family hypertrophic cardiomyopathy is more common in the U.S., while right ventricular arrhythmogenic dysplasia have a greater effect in the region of Veneto in Italy [15]. In Brazil, SCD was more frequent in soccer and basketball. The occurrence of benign or potentially malignant heart diseases cataloged over more than 30 years of evaluations of athletes ranging from adolescents to elderly veterans in various sporting modalities at Instituto Dante Pazzanese de Cardiologia, of children younger than 14 years old at social clubs in São Paulo, and of boys up to 18 years old who needed a "medical examination" to enable them to train professional soccer in São Paulo's major league clubs [16].

From 1966 to 2004 it was reported 1,101 cases of sudden death in athletes younger than 35 years, 90% were due to cardiovascular diseases, 50% presented anatomic congenital cardiac diseases and cardiomyopathies, 10% presented early coronary atherosclerotic disease, 40% occurred in athletes younger than 18 years and 33% below 16 years old, the ratio of female/male was 1/9. SCD was reported for almost all sports, the majority involved soccer (30%), basketball (25%) and runners (15%) [21].

Sadaniantz and Thompson [22] demonstrated that the relative risk for sudden death increases during exercise, although the absolute risk remains low. It is also demonstrated that individuals who practice physical activity less often present higher risk for sudden death compared to those who practice exercise regularly [23-27].

Jeresaty [28] and Sadaniatz et al [22] estimated that the prevalence of mitral valve prolapse was 6 to 17%. These authors reported that 3% of sudden deaths related to exercise are due to this dysfunction. If we consider this prevalence, the risk of sudden death caused by mitral valve prolapse is extremely low. If there are cases of syncope, chest pain, disabling, complex ventricular arrhythmias, cardiomegaly caused by moderate mitral valve regurgitation or sudden death in the family the exercise practice should be appropriately evaluated [22,28].

When we investigate the causes of sudden death in soccer players, we may describe two groups: The first includes congenital cardiac abnormalities (mainly obstructive hypertrophic cardiomyopathy and anomalous origin of coronary arteries), and the second due to coronary artery disease. In the both groups it is observed fatal arrhythmias (SCD).

### Pathophysiology

Several electrophysiological mechanisms may be responsible for SCD, however, in general it is resulted of complex interaction of factors such as arrhythmogenic substrate, regulator and triggers factors: arrhythmogenic substrate: it may be represented by a fibrotic or infiltrative myocardial process, myocardial necrosis, ventricular aneurysm, myocardial hypertrophy, myocardial inflammation and electrocellular changes; regulator factors: autonomic nervous system, renin-angiotensin system, hydro-electrolyte disturbances, hypoxia, drug action, hemodynamic factors, ischemia and blood pH and; trigger factors: extra-systole and abrupt increases of heart rate [15].

The arrhythmogenic substrates alone do not produce fatal arrhythmia. It is necessary trigger factors acting on it in a favorable context for the sustenance of the arrhythmia - modifying factors [20]. Over 90% of deaths among young athletes occur during training or competition [21,29,30]. It could support the fact that in the presence of certain diseases the physical exhaustion is the trigger of lethal arrhythmias and sudden death in sport area [20].

In general, the most common triggers for sudden death are excessive physical effort, stress and the transition period from sleep to wakefulness on the first hours of the morning. In these situations there is a peak of sympathetic activity which promotes sudden increase of myocardial demand and/or changes in vasomotor tone of coronary arteries. These conditions may lead to rupture of a plaque with subsequent thrombus formation, leading to myocardial ischemia which may be manifested as unstable angina, myocardial infarction or its complications [31]. Abrupt obstructions of blood flow to the heart may also result in arrhythmias and SCD. A sudden obstruction of the mitral valve by myxoma, aortic stenosis or obstruction of the left ventricle outflow by septal hypertrophy may provide a fatal event in an abrupt and unexpected way [31].

Although a rare event, sudden death in athletes generates great social and emotional impact throughout society, especially when the victim is a young athlete, it arises questions from the midia: Would be the sport detrimental to health? Sudden death could be prevented?

There is evidence that sports, including soccer, are healthy to body and mind, except for a small group of practitioners who have undiagnosed heart disease and which it would not be recommended. However, at least one of them could be prevented through pre-participatory evaluations recommended to all practitioners of physical activities in order to identify their conditions that would lead to the final event [32].

In all types of sports it has been considered older ages in which the athletes are classified as "masters". These ages are apparently arbitrary and vary according to the sports. For instance, in water polo the age is 25 years old and in bowling it is 50 years old. In soccer the age is 35 years old and it is not reported in the literature the objective criteria regarding these ages' limits. Similarly, the national and international publications follow this categorization and in the world all these ages are referred to as watershed between young athletes and "masters" [32].

### Cardiovascular causes of sudden death in athletes younger than 35 years old

For the vast majority of deaths caused by cardiovascular disease in athletes younger than 35 years old the literature relate congenital abnormalities of the heart, i.e., diseases at birth. Among them the most commons are: hypertrophic cardiomyopathy (36%), coronary artery anomalies (19%) and increased ventricular mass (10%). The remaining percentage is caused by other diseases such as right ventricular arrhythmogenic dysplasia, Marfan syndrome, the long QT syndrome, Brugada syndrome and catecholaminergic familiar tachycardia [33,34]. Unfortunately, effective detection of these diseases which may cause morbidity and sudden death in athletes is very low; it was reported combined prevalence of 0.2% [33,34].

### 1. Congenital cardiac anomalies

A large number of congenital cardiac anomalies may be found and listed as a cause of SCD during exercise; however, the most prevalent according to literature references are anomalous origin of left coronary artery from pulmonary artery and hypertrophic cardiomyopathy [34].

### 1.1 Origin of the left coronary artery from pulmonary artery trunk

It represents 0.25-0.5% or 4 per 1000 of all congenital heart disease [35], it occurs in 0.2-1.2% of the population and causes 12% of sudden deaths related to sports (and 1.2% of deaths not related to sports) [36]. Among all patients with anomalous origin of coronary arteries only 10% reach adulthood. The diagnosis is usually made post-death because most patients are asymptomatic and the aggressive surgical treatment is indicated due to the higher risk of SCD [37].

This pathological condition is not the only coronary anomaly that causes SCD. In fact, this entity is rather uncommon in adult population. The clinical and pathological profile may be divided into child and adult [36]. The child type is observed in the absence of collateral circulation, which explains the poor prognosis of patients without intervention and the pattern of myocardial infarction on electrocardiogram. It must cause in infants by unexplained cardiomegaly [38], suggesting that it is the responsibility of myocardial infarction, heart failure and possibly death during early childhood period [39]. The adult type is characterized by the absence of early symptoms. In this case the collateral circulation is present in a satisfactory manner and the electrocardiogram is not significantly changed or even normal. In this context coronary heart disease usually manifests during effort which explains the sudden deaths of young athletes [36].

The electrocardiogram (ECG) contributes to the most of clinical diagnosis achievement, especially in symptomatic infants. In children and adults the electrocardiogram may be normal or almost normal [40], however, two elements characterize the ECG of this entity: Standard of myocardial infarction, present in cases where the collateral circulation of the right coronary artery is insufficient (90% cases), more common in infants and; Left ventricular hypertrophy standard as a consequence of replication of myocytes conditioned by chronic hypoxia. It is more frequently observed in adults.

### 1.2. Hypertrophic cardiomyopathy

Hypertrophic cardiomyopathy may be defined as myocardial involvement without definitive etiology, characterized by left ventricle hypertrophy (rarely right ventricle), with myofibrillar disorder, increased left ventricular ejection fraction and decreased cardiac output, which depends on the functional impairment degree [41-47]. The outflow tract obstruction may exist or not. In this form of cardiomyopathy situations which present increased ventricular mass caused by hypertension, aortic stenosis or other defined causes are not included. According to the anatomical characteristics of hypertrophic cardiomyopathy it may be symmetrical or asymmetrical, in the case of SCD: asymmetric septal, ventricular mean, septal apical, posteroseptal or rarely of right ventricle [48].

The asymmetric septal hypertrophic cardiomyopathy is the most prevalent and may occur in non-obstructive or obstructive outflow of the left ventricle and it is revealed at rest or with stress. Hemodynamically, it is noted pressure gradient between the left ventricle and the aorta, resulted from septal hypertrophy, which avoids the flow through the left ventricle outflow. Due to the Venturi effect, during ventricular systole the anterior leaflet of the mitral valve come close to the interventricular septum (MSA) and anguishes the left ventricle outflow. The mitral valve becomes incompetent allowing blood reflux into the left atrial cavity due to the lacinia movement. Regardless of the obstruction, there is hipodiastole by impaired ventricular relaxation, which contributes to cardiac output decrease [48].

It is estimated that cardiomyopathy may affect between 0.001 and 0.2% of the population [49]. On the other hand, the occasional finding of some septal hypertrophy degree during echocardiography was estimated at approximately 0.5% [50]. It seems unanimous that hypertrophic cardiomyopathy is the leading cause of SCD related to exercises in athletes younger than 30 years old [51,52] which would be responsible for around half of deaths and often appearing as first and only manifestation of the disease. In some cases hypertrophy may be so important that septal thickness may reach 30 mm. This degree of hypertrophy possibly lead to left ventricular filling decrease and is able to produce severe hemodynamic changes in the left ventricle outflow, which may cause ischemia and potentially fatal arrhythmias [51].

Considering that septal hypertrophy may be totally asymptomatic, this disease may be not diagnosed. A detailed investigation in family history is of great value, since this disease has a pattern distribution of dominant autosomal genetic [53] in at least half of the cases, while the gene is located on chromosomes 1, 11, 14 or 15 [54] with predominance in males compared to females. Albeit the relationship between hypertrophy degree and symptoms magnitude is usually present, it is not always observed, since some patients have a significant degree of hypertrophy without any symptoms to report and the reverse situation also exists. However, the risk of sudden death is directly proportional to the left ventricular hypertrophy degree, and the annual incidence of sudden death is 0 to 1000 individuals with left ventricular thickness lower than 15 mm and 18.2 for 1000 patients with septal thickness equal or higher than 30 mm [55].

The natural history of hypertrophic cardiomyopathy leads to heart failure and/or sudden death. It is difficult to evaluate the prognosis of this disease because studies examined only symptomatic patients treated at major centers. Some manifestations are held for most of the authors as worse prognosis: early diagnosis (particularly children); large ventricular thickness, family history of sudden death, personal history of resuscitated sudden death, sustained (or not) ventricular tachycardia and exercise-induced hypotension [53-55].

### Causes of sudden death in athletes older than 35 years old

According to the literature, athletes older than 35 years old may die due to complications of coronary heart disease often caused by atherosclerosis. Myocardial ischemia and myocardial infarction are responsible for most of SCD above this age (80%) [51,56-60].

## Concluding Remarks

This review does not allow us to surely identify the preference causes of SCD in athletes. Although this etiology tends to prevail in older subjects, it seems that in competitive sports there is insufficient evidence regarding this matter. Acceptance of this premise may reduce the preventive approach to SCD just aiming to exclude congenital heart in young and just deal with coronary risk factors in the elderly, considering as secondary level the prevention of other possible etiologies for the athletes, including masters in football. The qualification by age implies that as a result of age the athletes would present limitations that would hamper their performance. Moreover, ages above 35 years old puts a real stigma in these athletes, thus, compromising physical performance, beyond the psychological involvement resulting on SCD risk.

## Competing interests

The authors declare that they have no competing interests.

## Authors' contributions

All authors participated in the revision of the manuscript. All authors conceived of the study, determined the design, performed the statistical analysis, interpreted the data and drafted the manuscript. All authors read and gave final approval for the version submitted for publication.
